# Educational impact of an active learning session with 6-lead mobile electrocardiography on medical students’ knowledge of cardiovascular physiology during the COVID-19 pandemic in the United States: a survey-based observational study

**DOI:** 10.3352/jeehp.2022.19.12

**Published:** 2022-06-20

**Authors:** Alexandra Camille Greb, Emma Altieri, Irene Masini, Emily Helena Frisch, Milton Leon Greenberg

**Affiliations:** 1 School of Medicine, University of California, Irvine, CA, USA; 2 Institute for Clinical and Translational Science, University of California, Irvine, CA, USA; 3 Department of Physiology and Biophysics, University of California, Irvine, CA, USA; Hallym University, Korea

**Keywords:** Cardiovascular physiological phenomena, COVID-19, Electrocardiography, Medical students, Active learning

## Abstract

Mobile electrocardiogram (ECG) devices are valuable tools for teaching ECG interpretation. The primary purpose of this follow-up study was to determine if an ECG active learning session could be safely and effectively performed during the coronavirus disease 2019 (COVID-19) pandemic using a newly developed mobile 6-lead ECG device. Additionally, we examined the educational impact of these active learning sessions on student knowledge of cardiovascular physiology and the utility of the mobile 6-lead ECG device in a classroom setting. In this study, first-year medical students (MS1) performed four active learning activities using the new mobile 6-lead ECG device. Data were collected from 42 MS1s through a quantitative survey administered in September 2020. Overall, students felt the activity enhanced their understanding of the course material and that the activity was performed safely and in compliance with local COVID-19 guidelines. These results emphasize student preference for hands-on, small group learning activities in spite of the pandemic.

## Background/rationale

The electrocardiogram (ECG) is a widely used diagnostic tool in medicine, and accurately interpreting ECGs is a critical skill that medical students must master. Graduating medical students are not adequately prepared to accurately assess ECGs in the clinical setting, and residents in specialties that frequently review ECGs often fail to recognize pathologic rhythms [1,2]. The AliveCor KardiaMobile, a telemedicine-appropriate, mobile ECG, is an effective tool for teaching ECG concepts to first-year medical students (MS1s) [3]. In 2019, AliveCor received regulatory approval for a new 6-lead KardiaMobile device. In this study, we developed a pandemic-appropriate active learning session to evaluate the educational utility of the new 6-lead ECG device.

Due to the coronavirus disease 2019 (COVID-19) pandemic, university guidelines restricted most face-to-face learning activities, excepting tactile-learning activities [4,5]. In-person medical education activities, including anatomy and clinical skills laboratories, were maintained in the pre-clerkship curriculum by utilizing personal protective equipment and limiting social interactions to pre-established clinical learning groups of 4–6 students each [4]. Currently, little is known about medical students’ self-perceptions of pandemic safety and knowledge acquisition in face-to-face learning activities during the COVID-19 pandemic.

## Objectives

This study aimed to assess students’ knowledge of cardiovascular physiology following the implementation of an active learning session using the new 6-lead ECG device. We hypothesized that the active learning session could be safely performed in person during the pandemic, would improve the teaching of cardiovascular physiology concepts, and increase familiarity with telemedicine-enabled ECG devices.

## Ethics statement

This study was qualified as exempt research by the University of California, Irvine, Institutional Review Board for Human Subjects. Responses to survey questions were anonymous, shared in aggregate form, and maintained privacy and confidentiality.

## Study design

We report an observational study based on a questionnaire survey after subjects had completed the active learning session. Reporting was consistent with the STROBE (Strengthening the Reporting of Observational Studies in Epidemiology) statement [6].

## Setting

This study was performed at the University of California, Irvine, School of Medicine in September 2020. There was no follow-up study. After completion of the active learning session, students were asked to complete an optional, anonymous survey via Qualtrics to understand participant perception of the ECG activity.

## Intervention, including general procedure and COVID-19 precautions

Prior to the active learning session, students were informed that the activity involved recording, interpretating, and sharing ECG readings with their fellow group members. Student participation was entirely optional, and no student was required to record and share their ECG reading with the group. Informed consent was obtained via Qualtrics survey for each participant. Faculty members and second-year medical students were available to review student ECG strips and advise further evaluation by a physician should any cardiovascular abnormality be detected during the active learning activity.

**Fig. 1.**
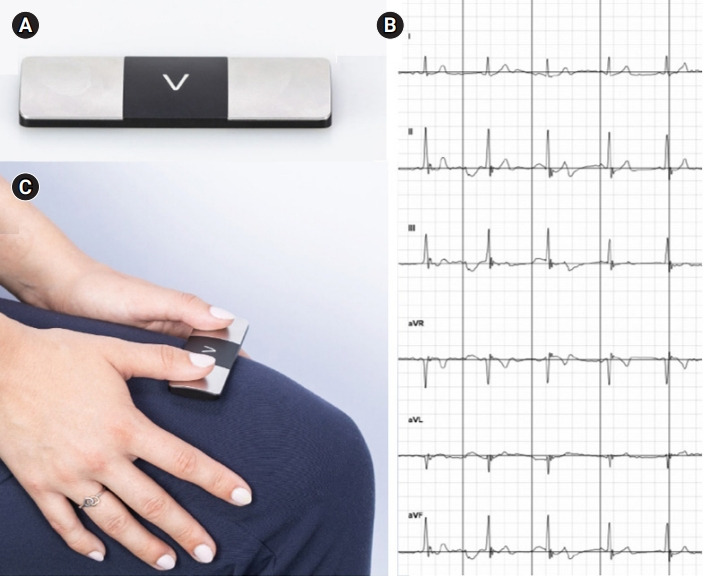
KardiaMobile equipment and output. (A) The KardiaMobile 6L electrocardiogram (ECG) medical device. (B) Example Kardia application output following an ECG recording. (C) Demonstration of how the device is placed on a participant to generate ECG recording data. Gain and paper speed are 10 mm/mV and 25 mm/ms.

KardiaMobile 6L devices and Apple iPads were provided to each student group (Fig. 1A). Instructions for the active learning activity, including example recordings (Fig. 1B) and guidance on ECG device placement (Fig. 1C), were also provided. All students completed the following four learning activities: “reading the ECG” to examine their classmates for common cardiac abnormalities that can be determined by an ECG recording; “comparing ECG readings” to examine common, non-pathological differences in their ECG recordings due to athletic training and body type; “calculating axis” to examine the frontal plane to calculate the electrical axis of the heart; and “autonomic regulation” to study the effects of vagal maneuvers and exercise on their ECG readings.

The active learning session took place in two lecture halls to abide by social distancing practices. The activity was split over two 50-minute periods to allow for smaller groups of approximately 25 students per session. Preestablished clinical learning groups were maintained to minimize new and unnecessary contact between participants. KardiaMobile ECG devices were cleaned with alcohol wipes between uses, and students were encouraged to use hand sanitizer before and after the active learning session. Within the lecture hall, small groups were physically distanced at least 6 feet (1.8288 m) apart and universal masking was enforced. Before the in-person session, students with a fever, cough, or any other COVID-19 symptoms were asked to self-triage and remain at home.

## Participants

Eligibility criteria included enrollment in the 1st-year medical physiology and pathophysiology course at the University of California, Irvine, School of Medicine. Exclusion criteria included students who had previously completed ECG training with different devices. Out of 49 target subjects, 46 students participated in the active learning session and survey.

## Variables

All 9 questionnaire items on knowledge, skills, and pandemic safety were variables following the 6-lead mobile ECG active learning session.

## Data source/measurement

We developed the 9 item, 4-point Likert scale survey instrument (Supplement 1) to evaluate students’ self-perceptions of knowledge, skills, and comfort with the active learning ECG activity. This instrument was designed following modification of the previously published active learning ECG survey [3]. Four additional survey questions related to the COVID-19 pandemic were included, which were modified from a previously published medical education COVID-19 survey [7], and we consulted faculty and students to establish face validity. Comments were adapted by the research group, using a similar process as described in prior studies involving medical student surveys [8], and no further changes were made to the adapted questionnaire. An internal reliability coefficient was calculated for the instrument after the questionnaire had been administered during the study to medical students who had recently received in person ECG training, and the instrument’s reliability was found to be very high (Cronbach α=0.98)(Dataset 1).

## Bias

Students self-selected to attend the active learning session and complete the post-activity survey were not assigned or chosen.

## Study size

The study size was not estimated because whole target students were recruited.

## Statistical methods

Descriptive statistics were used for the data analysis.

## Descriptive data for participants

Demographic data were not collected.

## Main results

**Fig. 2.**
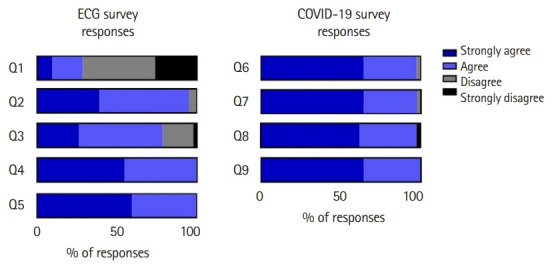
Post-activity survey responses assessing first-year medical students students’ evaluation of the electrocardiogram (ECG) active learning session. There were 42 responses, representing 91% of the students who attended the activity. Survey questions can be found in the “questionnaire survey” section. COVID-19, coronavirus disease 2019.

Student responses to the post-activity survey indicated that the majority of students had limited experiences with mobile medical devices before starting medical school (Fig. 2, Q1). Furthermore, 94% of students either agreed or strongly agreed that the AliveCor KardiaMobile device was a valuable addition to the “Reading ECG” session in Physiology (Fig. 2, Q2). Seventy-eight percent of participants agreed or strongly agreed that using the AliveCor KardiaMobile device helped further their understanding of ECGs (Fig. 2, Q3). All students agreed or strongly agreed that using mobile medical devices helped further their medical education (Fig. 2, Q4) and that mobile medical devices will be important in future clinical practice (Fig. 2, Q5).

Regarding student responses to holding an in-person active learning session during the height of the 2020 COVID-19 pandemic, 98% of students agreed or strongly agreed that they felt comfortable attending the “Reading ECG” session. They also felt that the “Reading ECG” session was operated in a manner that complied with current University COVID-19 guidance (Fig. 2, Q6, Q7). Regarding the in-person nature of the activity, 98% of students agreed or strongly agreed that the live ECG activity compared to a hypothetical virtual ECG activity, enhanced their understanding of the course material (Fig. 2, Q8). Every student agreed or strongly agreed that they preferred the live ECG activity compared to a hypothetical virtual ECG activity, to have the opportunity to interact with their classmates (Fig. 2, Q9). Raw data of participants’ response are available from Dataset 1.

## Interpretation

This follow-up study expands on the initial pilot study [3], using the newly developed 6-lead KardiaMobile devices in an in-person active learning ECG teaching session. In the current study, performed in 2020, 28% of students reported experience with mobile medical devices before starting medical school, compared with 22% in the previous survey from 2018 (Fig. 2, Q1). Students’ evaluation of the 2020 ECG activity is higher for every survey criterion than those in 2018, particularly for improving understanding of ECGs. This improvement in student satisfaction regarding the ECG teaching activity may be due to the new 6-lead device, inclusion of medical student peers in proctoring the session, physical interaction with peers during the pandemic, or other minor changes to the active learning session protocol.

## Comparison with previous studies

Active and experiential learning increases student engagement and competency [9], and the KardiaMobile ECG device is an active learning educational tool that supports the teaching of cardiovascular physiology [3]. The original single-lead device is currently integrated into the pre-clerkship curriculum in an active learning setting, where it serves as a basis for student-led review sessions and helps teach the basics of ECG interpretation [3,10]. University of California, Irvine physiology courses rapidly adjusted most course content to remote learning formats necessitated by the COVID-19 pandemic [4,11,12]. Based on the survey responses, nearly all the students that participated in the ECG activity felt comfortable being in person based on the activity’s compliance with school-mandated COVID-19 protocols (Fig. 1). Additionally, students felt that the in-person session was preferable over a hypothetical virtual session to interact with their classmates and learn essential ECG concepts, supporting the idea that medical students continue to prefer learning new material in an in-person setting [13]. Furthermore, previous studies demonstrate a decline in student performance with the shift to virtual learning during the pandemic, thus emphasizing the importance of safe and supportive in-person learning sessions in pre-clerkship medical training [14,15].

## Suggestion

Future directions for research in this area include incorporating the KardiaMobile device into remote ECG teaching modules during clinical rotations to emphasize basic pathophysiological concepts and to create a longitudinal learning experience between the 1st through 3rd years of medical school.

## Limitations

This study focuses on the interpretation of non-pathologic sinus rhythms, resulting in limited experience in reading abnormal ECG findings. In addition, we are not sharing a direct comparison to the single-lead KardiaMobile ECG device, so we cannot make any claims regarding if the 6-lead device provides a superior educational experience.

## Conclusion

The active learning with the 6-lead KardiaMobile ECG device is a valuable addition to physiology education for medical students. This study emphasizes student preference for hands-on, small group learning activities, particularly during social and educational isolation periods. Also, the hypothesis that the active learning session could be safely performed in person during the pandemic could be accepted.

## Footnotes

**Data availability**
Data files are available from Harvard Dataverse: https://doi.org/10.7910/DVN/U1TBMX Dataset 1. Raw response data files.

## Supplementary materials

Data files are available from Harvard Dataverse: https://doi.org/10.7910/DVN/U1TBMX

Supplement 1. Items of the survey questionnaire.

Supplement 2. Audio recording of the abstract.
