# Supplementary material for: Educational impact of an active learning session with 6-lead mobile electrocardiography on medical students’ knowledge of cardiovascular physiology during the COVID-19 pandemic in the United States: a survey-based observational study
Source: J Educ Eval Health Prof. 2022 Jun 20;19:12. doi: 10.3352/jeehp.2022.19.12 (PMC9343237; doi:10.3352/jeehp.2022.19.12)
Supplement: Supplementary file 2 — Supplement 1. Items of the survey questionnaire. [file jeehp-19-12-suppl.docx]

**Supplement 1.** Items of the survey questionnaire.

**Survey questions:**

Q1) I had experience with mobile medical devices before starting medical school.

Q2) The AliveCor KardiaMobile device was a valuable addition to the “Reading electrocardiogram (ECG)” session in Physiology.

Q3) I felt that using the AliveCor KardiaMobile device helped further my understanding of ECGs.

Q4) I feel that using mobile medical devices will help further my medical education.

Q5) Knowing about mobile medical devices is important in my future practice as a physician.

Q6) I felt comfortable attending the “Reading ECG” session despite the ongoing coronavirus disease 2019 (COVID-19) pandemic.

Q7) I felt that the “Reading ECG” session was operated in a manner that complied with current University COVID-19 guidance.

Q8) I preferred the live ECG activity over a hypothetical virtual ECG activity to enhance my understanding of the course material.

Q9) I preferred the live ECG activity over a hypothetical virtual ECG activity to have the opportunity to interact with my classmates.
